# Supplementary material for: The immunoproteasome‐specific inhibitor ONX 0914 reverses susceptibility to acute viral myocarditis
Source: EMBO Mol Med. 2018 Jan 2;10(2):200–18. doi: 10.15252/emmm.201708089 (PMC5801517; doi:10.15252/emmm.201708089)
Supplement: Supplementary file 1 — Expanded View Figures PDF [file EMMM-10-200-s001.pdf]

Expanded View Figures

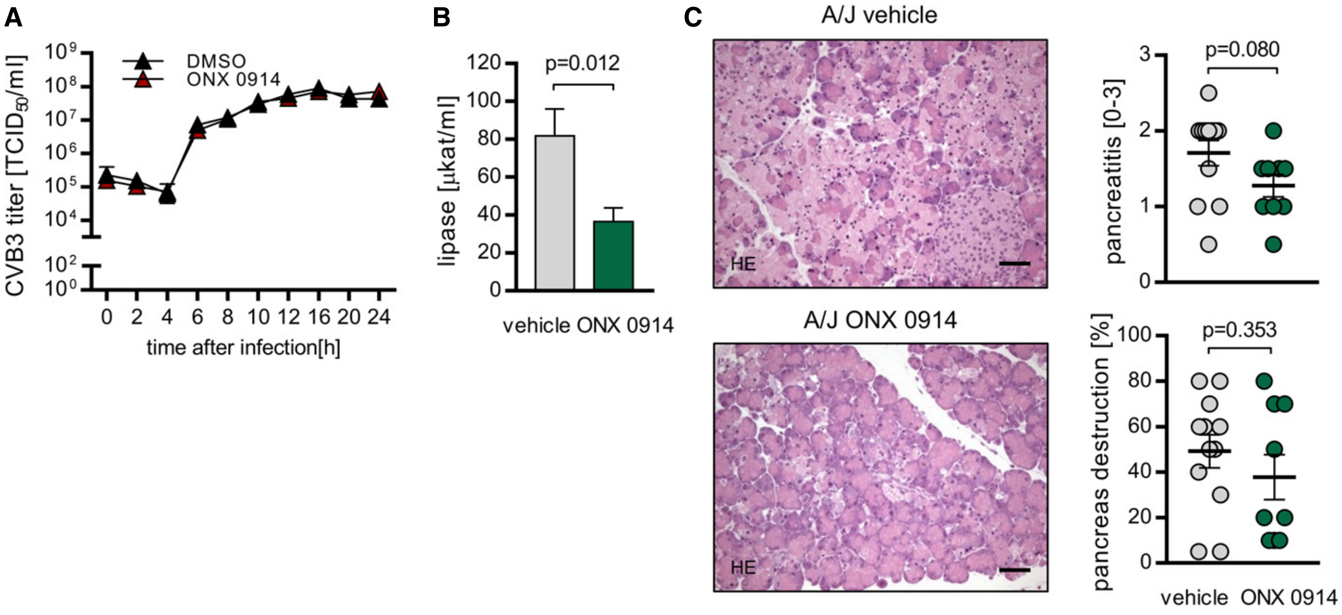

Figure EV1. ONX 0914-induced effects on viral replication and pancreatic tissue damage.

A Since ERK1/2 signaling is crucial for proper CVB3 replication in cardiac cells (Luo *et al*, 2002) and ONX 0914 negatively influences ERK1/2-phosphorylation (Fig 8), we considered it possible that ONX 0914 exerts direct antiviral effects thereby contributing to the reduction in myocardial viral burden observed early during CVB3 infection of inhibitor-treated animals. To clarify whether ONX-0914 might influence different phases of the viral life cycle, we generated one-step growth curves for embryonic cardiomyocytes as representative targets for infection of heart muscle cells *in vivo*. ONX 0914 was used *in cellulo* at 75 nM—a concentration verified for maintained cell viability and LMP7-specific inhibition (Spur *et al*, 2016). Primary embryonic cardiomyocytes were treated with ONX 0914 for 3 h. Simultaneously, cells were infected with CVB3 (MOI 5). After the indicated time points p.i., amount of infectious virus of total cell cultures (in triplicate per time point) was determined by $TCID_{50}$ assay. All data are means ± SEM. In the presence of ONX 0914, we could observe unhindered replication of CVB3.

B, C A/J mice were compound-treated starting one day prior to CVB3 infection. Two days after viral infection, the level of infectious virus particles peaked in pancreas yielding no differences between vehicle- and compound-treated mice. To investigate whether ONX 0914 might influence tissue injury of exocrine pancreatic cells, serum activity of lipase (B) was determined by automatic procedures (mice from a total of four separate experiments; vehicle n = 7, ONX 0914 n = 8; unpaired t-test). Pancreas inflammation was scored on a scale from 0 to 3 with 0 indicating no inflammation and three indicating severe inflammation. Two representative HE stains are depicted. Scale bar indicates 60 µm. Also, pancreas destruction was quantified histologically (mice from a total of four separate experiments; vehicle n = 12, ONX 0914 n = 9; Mann–Whitney test). All data are means ± SEM.

© 2018 The Authors

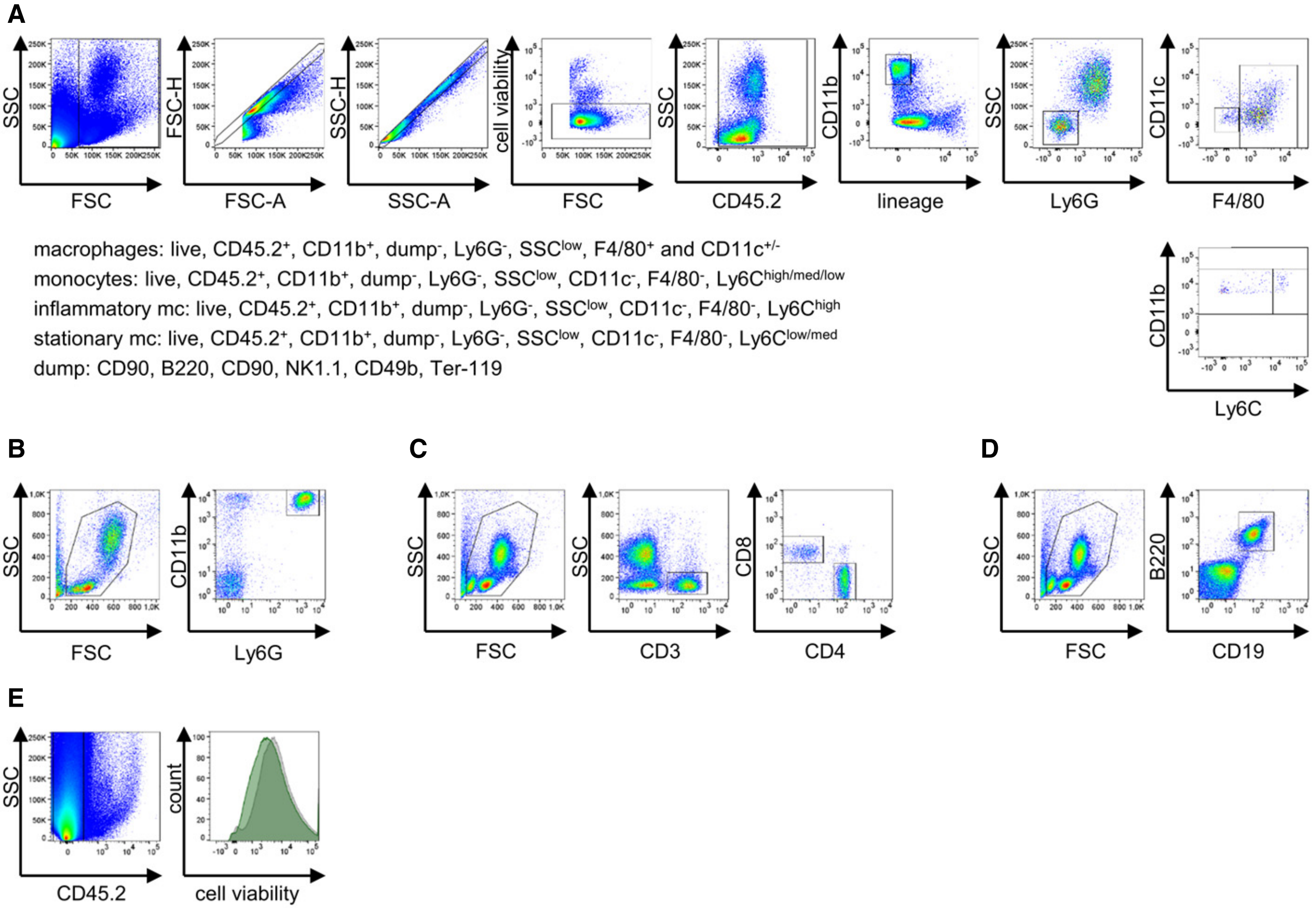

Figure EV2. Gating strategy for the different immune cell populations after flow cytometry.

Gating strategy for the different immune cell populations after flow cytometry is depicted.

A Myeloid cell characterization strategy (LSR II machine). Cells were first gated on size and singularity followed by viability dye exclusion to identify live cells for further analysis. Live cells were gated on the expression of CD45 and further of CD11b to identify myeloid cells. Finally, non-neutrophil (Ly6G^{-}) myeloid cells were discriminated additionally by assessing expression of F4/80 and Ly6C. Monocytes were identified as Fixable Viability Dye^{low}, CD45.2^{+}, CD11b^{high}, lineage (B220, CD90.2, CD49, NK-T/NK Cell Antigen, Ter-119)$^{-}$, Ly6G^{-}, SSC^{low}, F4/80^{-}/CD11c^{-}, and further differentiated according to Ly6C expression: Inflammatory monocytes are Ly6C^{high} and patrolling monocytes are Ly6C$^{med/low}$. Macrophages: Fixable Viability Dye^{low}, CD45.2^{+}, CD11b^{high}, lineage^{-}, Ly6G^{-}, SSC^{low}, F4/80^{+}/CD11c$^{low/+}$. Neutrophils: Fixable Viability Dye^{low}, CD45.2^{+}, CD11b^{high}, lineage^{-}, Ly6G^{+}, SSC^{high}.

B Neutrophil characterization strategy (FACS Calibur machine). Cells were first gated on size as described above. Live cells were then gated on co-expression of CD11b and Ly6G.

C T-cell characterization strategy (LSR II machine). Cells were first gated on size, singularity, and viability as described above. Live cells were then gated on co-expression of CD3 and CD4 or CD8, respectively. T lymphocytes: Fixable Viability Dye^{low}, CD45.2^{+}, B220^{-}, CD3^{+}, and either CD4^{+} or CD8^{+}.

D B-cell characterization strategy (LSR II machine). Cells were first gated on size, singularity, and viability as described above. Live cells were then gated on co-expression of CD19 and B220. B lymphocytes: Fixable Viability Dye^{low}, CD45.2^{+}, CD3^{-}, B220^{+}/CD19^{+}.

E Cell death in non-immune cardiac cells. Cells were first gated by CD45.2^{+} exclusion to include all cardiomyocytes. Median fluorescence intensity (MFI) of Fixable Viability Dye eFluor 780 was determined in vehicle- and ONX 0914-treated mice. One representative histogram is depicted.

© 2018 The Authors

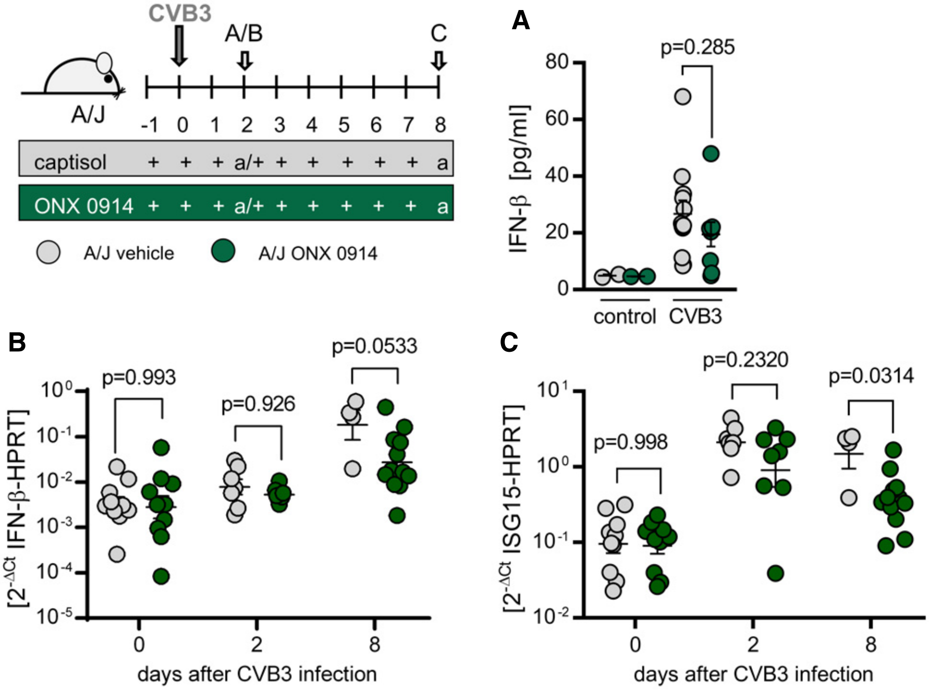

Figure EV3. Induction of T1IFN response in A/J mice under ONX 0914 influence during CVB3 infection.

A–C A/J mice were infected with 10^4 PFU CVB3 (Nancy). ONX 0914 or vehicle treatment was carried out daily, starting one day prior to virus inoculation. IFN-β serum levels during early infection (d2 p.i.) were determined by ELISA (control = uninfected animals) (control *n* = 2 per group, vehicle *n* = 12, ONX 0914 *n* = 9) (A). At days 2 and 8 p.i., total heart tissue mRNA was isolated, reverse transcribed, and IFN-β (B) as well as ISG15 (C) mRNA expression was determined by TaqMan qPCR (vehicle *n* = 10, 8, 4, ONX 0914 *n* = 10, 8, 12 for day 0, 2 and 8 respectively). Data are mean ± SEM. *P*-values are indicated in each graph; unpaired *t*-test was conducted.

 © 2018 The Authors

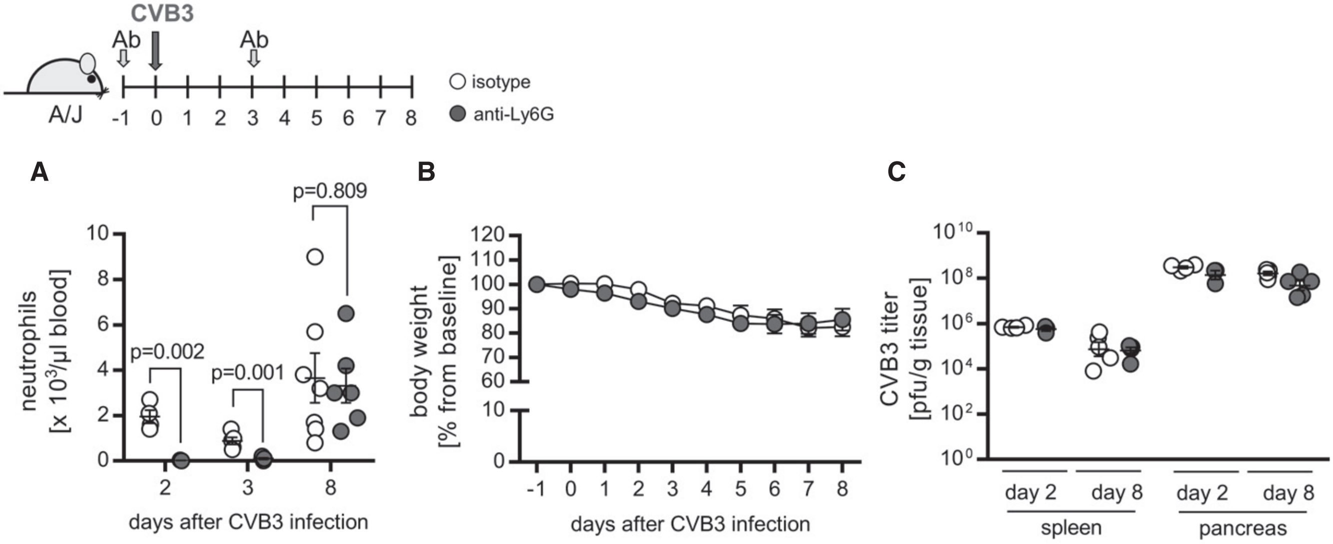

Figure EV4. Impact of neutrophil depletion during CVB3 infection.

A–C Neutrophil depletion was achieved by administration of antibodies directed against Ly6G one day prior to CVB3 infection (3.3×10^3 PFU). For a 2-day experiment, Ly6G antibody injection was repeated at day 1 p.i. For mice that were sacrificed at day 8, antibody injection was repeated after 4 days (3-day p.i.). Peripheral neutrophil abundance was determined by automatic blood counting (A). Unpaired *t*-tests were conducted. *P*-values are indicated. Body weight was monitored for an 8-day period. For the indicated time points p.i., average percentage of weight loss relative to the initial value ± SEM is shown (B). Viral load in spleen and pancreas was determined by standard plaque assay assessing the amount of infectious virus particles (C). (isotype n = 4, 5, 10, anti-Ly6G n = 3, 5, 6 for day 2, 3 and 8 respectively). Transformed means ± SEM are presented.

Figure EV5. Ubiquitin conjugate formation and lack of significant apoptotic cell death under ONX 0914 influence in A/J mice.

A To visualize the abundance of ubiquitinated proteins during myocarditis in A/J mice, immunohistology staining of ubiquitin was performed on heart tissue sections during acute myocarditis (day 8 p.i.) according to established procedures (Opitz *et al*, 2011). Slides from four different vehicle and ONX 0914-treated mice are depicted. Positive signals were detected in both groups predominantly within inflammatory foci with a clear tendency toward increased signal intensity in vehicle-treated mice. Scale bar: 120 µm.

B Since inflammation in B6-LMP7$^{-/-}$ provoked apoptotic cell death, we questioned whether ONX 0914 treatment during myocarditis induces similar effects in A/J mice. Apoptosis was assessed on heart tissue sections obtained from CVB3-infected A/J mice (day 8 p.i.) by *in situ* cell death detection kit, TMR red (TUNEL assay) as described elsewhere (Opitz *et al*, 2011; Paeschke *et al*, 2016). Sections depicting inflammatory lesions are illustrated for n = 3 vehicle- and ONX 0914-treated mice. Other than revealed in DNase-treated samples, neither ONX 0914 treatment, CVB3 infection nor both conditions resulted in significant apoptotic cell death in heart muscle. Scale bar: 100 µm.

▶

© 2018 The Authors

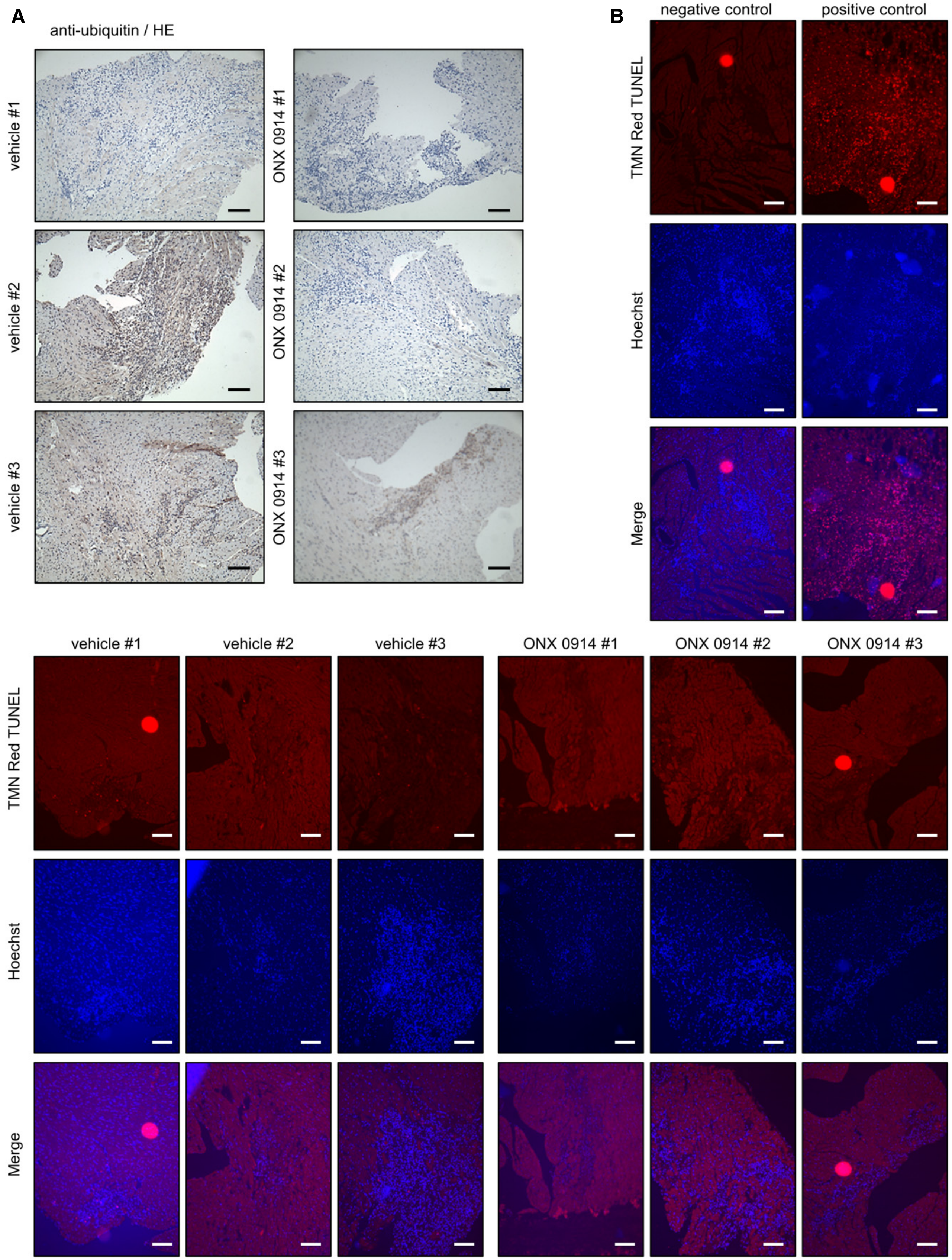

Figure EV5.

© 2018 The Authors
