# Supplementary material for: The immunoproteasome‐specific inhibitor ONX 0914 reverses susceptibility to acute viral myocarditis
Source: EMBO Mol Med. 2018 Jan 2;10(2):200–18. doi: 10.15252/emmm.201708089 (PMC5801517; doi:10.15252/emmm.201708089)
Supplement: Supplementary file 3 — Source Data for Figure 5G [file EMMM-10-200-s002.pptx]

## Slide 1
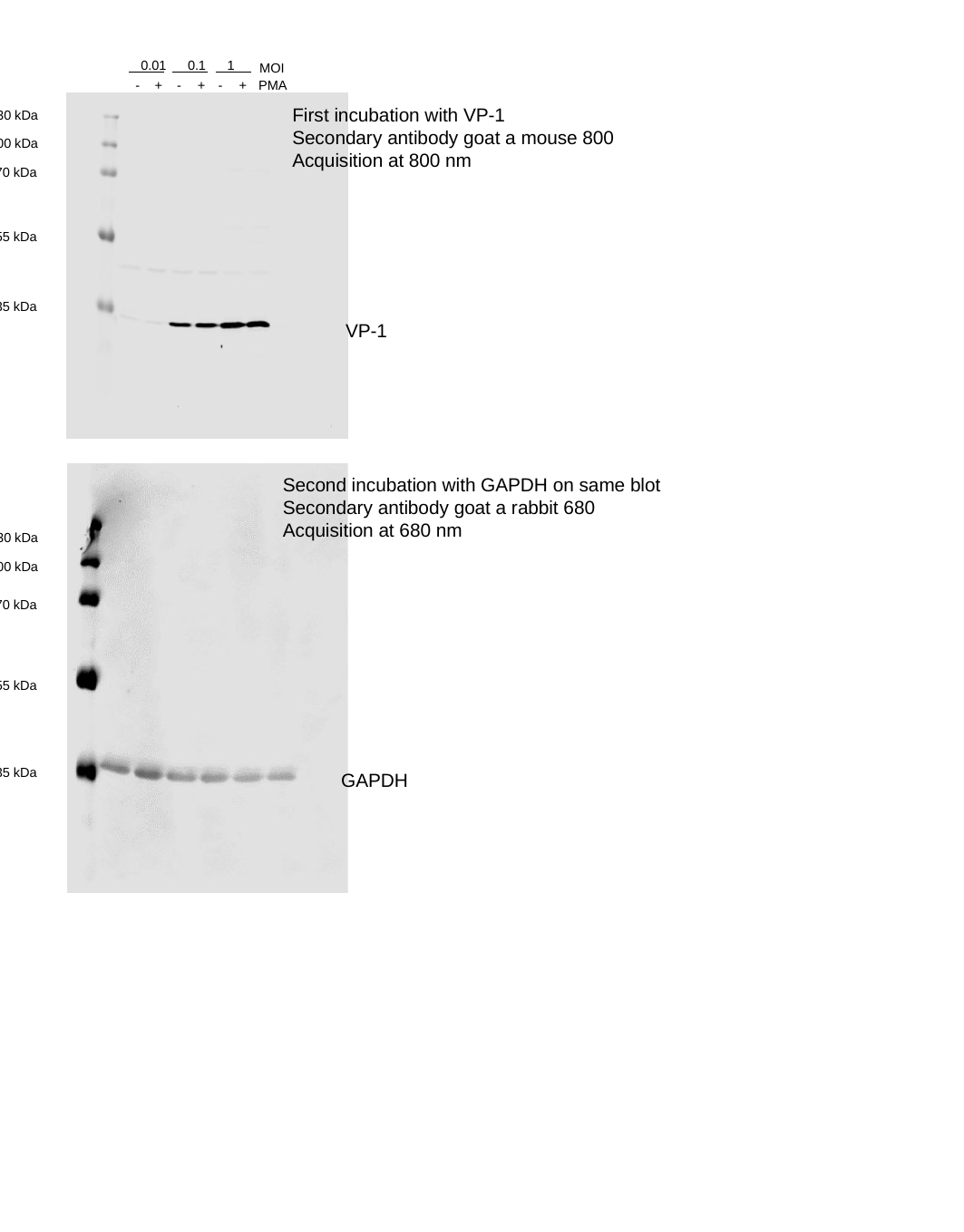

0.01
0.1
1
MOI
-
+
-
+
-
+
PMA
First incubation with VP-1
Secondary antibody goat a mouse 800
Acquisition at 800 nm
130 kDa
100 kDa
70 kDa
55 kDa
35 kDa
VP-1
Second incubation with GAPDH on same blot
Secondary antibody goat a rabbit 680
Acquisition at 680 nm
130 kDa
100 kDa
70 kDa
55 kDa
35 kDa
GAPDH
