# Supplementary material for: The immunoproteasome‐specific inhibitor ONX 0914 reverses susceptibility to acute viral myocarditis
Source: EMBO Mol Med. 2018 Jan 2;10(2):200–18. doi: 10.15252/emmm.201708089 (PMC5801517; doi:10.15252/emmm.201708089)
Supplement: Supplementary file 4 — Source Data for Figure 8C [file EMMM-10-200-s003.pptx]

## Slide 1
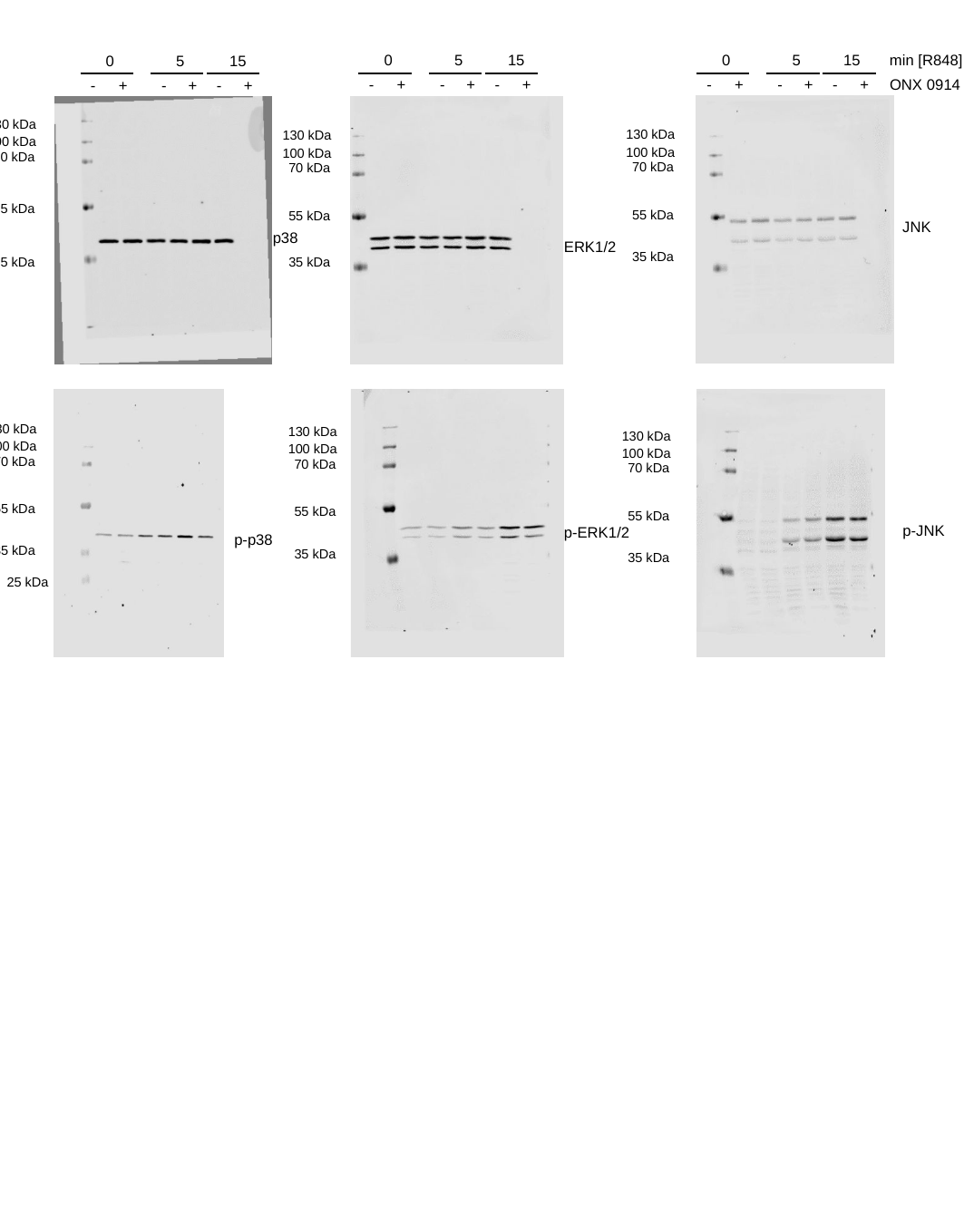

0
5
15
0
5
15
min [R848]
0
5
15
-
+
-
+
-
+
-
+
-
+
-
+
ONX 0914
-
+
-
+
-
+
130 kDa
130 kDa
130 kDa
100 kDa
100 kDa
100 kDa
70 kDa
70 kDa
70 kDa
55 kDa
55 kDa
55 kDa
JNK
p38
ERK1/2
35 kDa
35 kDa
35 kDa
130 kDa
130 kDa
130 kDa
100 kDa
100 kDa
100 kDa
70 kDa
70 kDa
70 kDa
55 kDa
55 kDa
55 kDa
p-JNK
p-ERK1/2
p-p38
35 kDa
35 kDa
35 kDa
25 kDa
